# Nevada’s Healthcare Crisis: A Severe Shortage of Physicians and Residency Positions

**DOI:** 10.7759/cureus.41700

**Published:** 2023-07-11

**Authors:** Kenny Do, Jenifer Do, Eric Kawana, Ren Zhang

**Affiliations:** 1 Kirk Kerkorian School of Medicine at UNLV (University of Nevada, Las Vegas), University of Nevada, Las Vegas, Las Vegas, USA; 2 School of Life Sciences, University of Nevada, Las Vegas, Las Vegas, USA; 3 Colorectal Surgery, Nevada Surgery and Cancer Care, Las Vegas, USA

**Keywords:** specialty treatment, prevention in primary care, medical residency, physician shortage, global healthcare systems

## Abstract

The state of Nevada is home to millions of people and a prominent entertainment industry. However, the state ranks among the lowest in terms of available primary care doctors and general surgeons per capita, resulting in limited access to essential healthcare services and an increased reliance on emergency departments and hospitals. Nevada also faces the challenges posed by an aging physician workforce and a significant proportion of inactive providers. The scarcity of residency positions in Nevada's medical schools drives many graduates to seek residency training opportunities elsewhere, leading to a reduced likelihood of their return to practice within the state. We propose potential solutions, including increased funding for residency positions, prioritizing the retention of medical school graduates through local residency training, and the establishment of interdisciplinary comprehensive academic health centers. These measures are essential to meet the escalating healthcare demands of Nevada's rapidly growing population and to ultimately enhance patient outcomes.

## Editorial

With a population of over 3 million people and with one of the biggest entertainment capitals in the world, Las Vegas, the state of Nevada still faces a healthcare crisis [1]. Out of the 50 states in America, Nevada is currently ranked forty-eighth for the number of primary care doctors available for 100,000 Nevadans and ranked forty-ninth for the number of general surgeons available for 100,000 Nevadans [2]. The state needs an additional 2,561 physicians in its healthcare workforce just to meet the country’s standards. Recent reports have suggested that the state needs 540 additional surgeons, 1,038 physicians in medical specialties, and many more physicians from other fields [3].

Current research has shown that there is a higher probability of a healthy community when there are more primary care physicians available for the general population. Concurrently, an increase in the number of primary care physicians has also been shown to lower the frequency of patients presenting to the emergency department or hospital [4]. One study assessed the association between the severity of cardiovascular disease and access to primary care. It concluded that the readmission rate for cardiovascular patients was 33.3% for those who had challenges receiving preventative care, which is significantly higher than the 17.9% readmission rate for patients who had access to primary care [5]. For patients in Nevada, many may wait up to or even longer than one month just to see their primary care physicians. For specialty care, the wait time may even be more extended, and this is assuming that the specialty exists in the state.

The state also has a problem with an aging physician workforce and a high number of inactive providers. Close to 17% of the licensed doctors in Nevada are inactive, and these are providers who are retired or are not currently practicing medicine [3]. The growing age of the providers in Nevada poses another problem to the physician shortage in the state. In some of Nevada’s counties, the average age of allopathic doctors is 61.2 and the average age of osteopathic doctors is 57.7. These numbers are higher than the national averages of 52.9 for allopathic and 49.7 for osteopathic physicians, respectively [3].

One of the reasons for the shortage of medical specialties and care in the state is the lack of residency spots in its academic health systems. Currently, there are two allopathic and one osteopathic medical school in Nevada. The Kirk Kerkorian School of Medicine at UNLV (University of Nevada, Las Vegas) has a class size of 66, the University of Nevada Reno (UNR) School of Medicine has a class size of 70, and Touro University has a class size of 180. Ideally, these three schools should generate more than 300 physicians each year. However, this is not the case because of the limited residency spots in Nevada. This means that eventually, most of these graduates end up leaving the state and completing residency elsewhere [6]. To make matters worse, studies have shown that the number of physicians who eventually reside within a 100-mile radius of where they completed residency can be up to 50%, meaning that many of the Nevada medical school graduates may not even come back to practice in the state [6].

As of now, there are around 404 residency positions that are funded by the Center for Medicare and Medicaid Services (CMS) in the state [6]. In the past two residency match cycles, only 14.6% and 36.2% of the residency positions in Nevada were filled by UNR and UNLV medical students, respectively [6]. It is likely that these students have to leave the state for residency training because many specialties and subspecialties are missing from Nevada’s graduate medical education. There are no residency or fellowship programs for dermatology, ophthalmology, neurosurgery, urology, hematology/oncology, and rheumatology [6]. To keep physicians in Nevada, it is not enough just to recruit medical students who have close ties to the state. Although there is a 30% chance that these students will practice in Nevada, this number increases to more than 70% if the same students complete their residency in the state as well [7]. With Nevada being the fifth fastest-growing state in the country, it is crucial that the state gets more funding for residency and fellowship positions to address the increasing healthcare needs of its growing and aging populations [8].

Nevada is currently in the works of building major academic health centers. Currently, it has UNLV Health, which was first founded in 2017 and is growing to become a major academic health system that provides care to the state’s southern populations [9]. Studies have shown that academic teaching hospitals and their affiliated medical schools are at the forefront of conducting research to find novel treatments for diseases and improving methods to yield the best patient outcomes. In fact, the National Institutes of Health (NIH) funds over half of its external medical research for these academic health centers [10]. Other studies have found that patients who are treated at these academic health centers have better outcomes than if they are seen at nonteaching hospitals [10]. Not only should the state build a major academic health center that possesses all major medical specialties, but it should also work concurrently with other disciplines such as dentistry, nursing, occupational therapy, and physical therapy [9]. This creates an interconnected health network that ensures patients receive comprehensive and convenient care.

Conclusion

Nevada’s shortage of physicians should be addressed immediately, considering that the state’s population is growing at a rapid rate. One of the ways to address this issue is to increase residency positions and specialties because physicians who train in the state are more likely to stay and serve the local communities, especially if they completed medical school here as well. Increasing the number of physicians in the state would not only yield better patient outcomes, but it would also diversify Nevada’s economy by creating more jobs in its healthcare field.

## References

[1] (2023). U.S. Census Bureau QuickFacts: Nevada. https://www.census.gov/quickfacts/NV.

[2] Bush J (2023). Rosen introduces package of bipartisan bills to address doctor shortage in Nevada. Jacky Rosen.

[3] (2023). Physician numbers are on the rise, though Nevada still well below the national average. https://thenevadaindependent.com/article/physician-numbers-are-on-the-rise-though-nevada-still-well-below-the-national-average.

[4] Shi L (2012). The impact of primary care: a focused review. Scientifica (Cairo).

[5] Dupre ME, Xu H, Granger BB (2018). Access to routine care and risks for 30-day readmission in patients with cardiovascular disease. Am Heart J.

[6] (2023). Medical school, researchers call attention to NV’s shortage of doctor residency programs. https://www.nevadacurrent.com/2023/02/03/medical-school-researchers-call-attention-to-nvs-shortage-of-doctor-residency-programs/.

[7] (2023). Residency slots key to fixing physician shortage in Nevada. https://www.unlv.edu/news/article/residency-slots-key-fixing-physician-shortage-nevada.

[8] (2023). Nevada was the fifth-fastest growing state over the last decade according to new census data. https://thenevadaindependent.com/article/nevada-was-the-fifth-fastest-growing-state-over-the-last-decade-according-to-new-census-data.

[9] (2023). UNLV Medicine becomes UNLV Health. https://www.unlv.edu/news/article/unlv-medicine-becomes-unlv-health.

[10] (2023). Academic health centers save millions of lives. https://www.aamc.org/news/academic-health-centers-save-millions-lives.

